# Machine Learning Approach to Extract Diagnostic and Prognostic Thresholds: Application in Prognosis of Cardiovascular Mortality

**DOI:** 10.1155/2012/750151

**Published:** 2012-08-09

**Authors:** Luis J. Mena, Eber E. Orozco, Vanessa G. Felix, Rodolfo Ostos, Jesus Melgarejo, Gladys E. Maestre

**Affiliations:** ^1^Department of Computer Engineering, Polytechnic University of Sinaloa, 82199 Mazatlan, SIN, Mexico; ^2^Institute for Biological Research and Cardiovascular Institute, Faculty of Medicine, University of Zulia, Maracaibo 4002, Venezuela; ^3^Departments of Psychiatry and Neurology, and the Gertrude H. Sergievsky Center, Columbia University, New York, NY 10032, USA

## Abstract

Machine learning has become a powerful tool for analysing medical domains, assessing the importance of clinical parameters, and extracting medical knowledge for outcomes research. In this paper, we present a machine learning method for extracting diagnostic and prognostic thresholds, based on a symbolic classification algorithm called REMED. We evaluated the performance of our method by determining new prognostic thresholds for well-known and potential cardiovascular risk factors that are used to support medical decisions in the prognosis of fatal cardiovascular diseases. Our approach predicted 36% of cardiovascular deaths with 80% specificity and 75% general accuracy. The new method provides an innovative approach that might be useful to support decisions about medical diagnoses and prognoses.

## 1. Introduction

Machine learning (ML) disciplines provide computational methods and learning mechanisms that can help generate new knowledge from large databases. Applications of ML are useful for constructing approaches to solving problems of classification, prediction, recognition patterns, and knowledge extraction, where the data take the form of a set of examples, and the output takes the form of prediction of new examples [1, 2]. In this sense, ML can provide techniques and tools that help solve diagnostic and prognostic problems in medical domains, where the input is a dataset with characteristics of the subjects, and the output is a diagnosis or prognosis of a specific disease [3]. Although diagnosis and prognosis are relatively straightforward ML problems, clinical decision-making using ML applications is not yet widely used by the medical community [4], because such a complex task requires not only accuracy, but also the confidence of physician specialists about the functional use of ML approaches in the medical field.

To successfully implement an ML application in problems related to clinical decisions, it is necessary to consider some specific requirements [4, 5]. For example, the prediction of disease progression is generally associated with the evolution of certain risk factors; in the case of some chronic diseases (e.g., cancer, cardiovascular diseases, and diabetes), the risk factors include nonchangeable characteristics, such as age or gender. The use of such nonchangeable qualities to predict the onset of a disease might not be as useful for avoiding evolution of the disease, because currently there is no medical treatment for modifying these biological characteristics. Thus, ML applications usually focus on changeable qualities, which make the prognostic task more difficult and complex.

Another important aspect to consider is the need to obtain interpretable approximations, in order to provide medical staff with useful information about the given problem. This is typically achieved using symbolic learning methods (e.g., decision trees and rules systems), which allow decisions to be explained in an easily comprehensible manner. However, the use of a symbolic learning algorithm to obtain a more comprehensible model frequently sacrifices accuracy in the prediction.

Another problem that often hinders high overall performance in the analysis of medical datasets is that generally these exhibit an unbalanced class distribution [6], which include a majority or negative class of healthy people (normal data) and a minority or positive class of sick people (the important class) with higher cost of erroneous classification. The latter usually has a higher rate of misclassification, because the performance of standard ML algorithms tends to be overwhelmed by the majority class, ignoring the minority class examples and obtaining results with acceptable accuracy and specificity (healthy subjects diagnosed correctly), but low sensitivity (sick subjects diagnosed correctly).

In addition to developing ML approaches that result in good overall performance and provide medical staff with interpretable prognostic information, providing the ability to support decisions and to reduce the number of medical tests for a reliable prognosis are also desirable. A measure of reliability of the diagnosis or prognosis is also important, because this would give medical staff sufficient confidence to put the new approach into practice. On the other hand, it is also desirable to have an approach that can provide reliable predictions based on a small amount of information about the patient, because collection of that information is often expensive, possibly subject to privacy issues, time consuming, and possibly harmful to the patient [4].

The present study focused on the implementation of a ML method to support medical decisions in the prognosis of fatal cardiovascular diseases, which are ranked among the top ten in the global disease burden [7]. The goal was to solve previously identified problems, through interdisciplinary work that included the collection and preprocessing of data from an ambulatory blood pressure (ABP) monitoring study [8], the implementation of a current ML algorithm with specific application to medical diagnosis and prognosis [9], and the identification of new prognostic thresholds for risk factors of cardiovascular mortality.

## 2. Methods

### 2.1. Ambulatory Blood Pressure Monitoring

**Figure 1:**
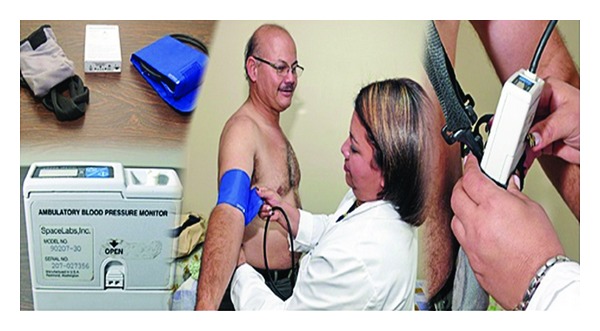
Ambulatory blood pressure monitoring procedure.

Currently available ABP monitors are fully automatic and portable devices (Figure 1) that can record BP for 24 hours or longer, while patients go about their normal daily activities [10]. This BP measurement technique provides a better estimate of risk in an individual patient than the traditional method, because it removes variability among individual observers, avoids the “white coat” effect (the transient but variable elevation of BP in a medical environment) [11] and the “masked hypertension” (normotensive by clinic measurement and hypertensive by ambulatory measurement) [12] and includes the inherent variability of BP [13]. Detailed descriptions of the ABP measurement methods are provided in previous reports of the Maracaibo Aging Study (MAS) [8, 14, 15].

### 2.2. Subjects

The MAS is an ongoing population-based, longitudinal study that includes 2500 subjects older than 55 years, residing in the Santa Lucia County, Maracaibo, Venezuela. All participants underwent extensive clinical and laboratory examinations and randomly selected individuals also underwent ABP monitoring. Informed consent was obtained from the subjects who agreed to participate, and from a close family member when doubts existed about the competence of the subject. The ethical review board of the Institute of Cardiovascular Diseases of the University of Zulia approved the protocol.

### 2.3. Cardiovascular Risk Factors

The leading global risk factor for mortality is high BP, which is responsible for 13% of deaths globally. Eight changeable risk factors (alcohol use, tobacco use, high BP, high body mass index, high cholesterol, high blood glucose, low fruit and vegetable intake, and physical inactivity) account for 61% of cardiovascular deaths. Combined, these same risk factors account for over three quarters of ischaemic heart disease, the leading cause of death worldwide [16].

However, investigators continue to look for new and emerging risk factors for cardiovascular disease. Recent ABP monitoring studies using a novel variability index [14] reported significant relationships between high BP variability (BPV) and cardiovascular outcomes [17–19]. BPV is a multifaceted phenomenon, influenced by the interaction between external emotional stimuli, such as stress and anxiety, and internal cardiovascular mechanisms that can vary from heartbeat to heartbeat. However, the complexity of BPV makes analysis difficult, and its independent contribution as a predictor of cardiovascular outcomes is not yet clear [20]. The present study aimed to identify new prognostic thresholds of risk factors for cardiovascular mortality, including high BP (the most significant cardiovascular predictor) and abnormal BPV (a potential independent predictor).

To estimate 24-hour BP level, we computed the weighed mean of valid BP readings (WBP) using the time interval between successive valid measurements as weighting factors [18]. In the case of BPV over 24 hours, we calculated the Average Real Variability (ARV) index [14] using (1):

(1) $$\begin{array}{c} \text{ARV}=\frac{1}{\sum w_{k}}\sum_{k=1}^{n-1}w_{k}\times \left|\text{B}{\text{P}}_{k}-\text{B}{\text{P}}_{k-1}\right|, \end{array}$$

where *n* is the number of valid BP readings, *k* ranges from 1 to *n*−1, and *w*_*k*_ is the time interval between BP_*k*_ and BP_*k*−1_.

### 2.4. Machine Learning Approach

**Figure 2:**
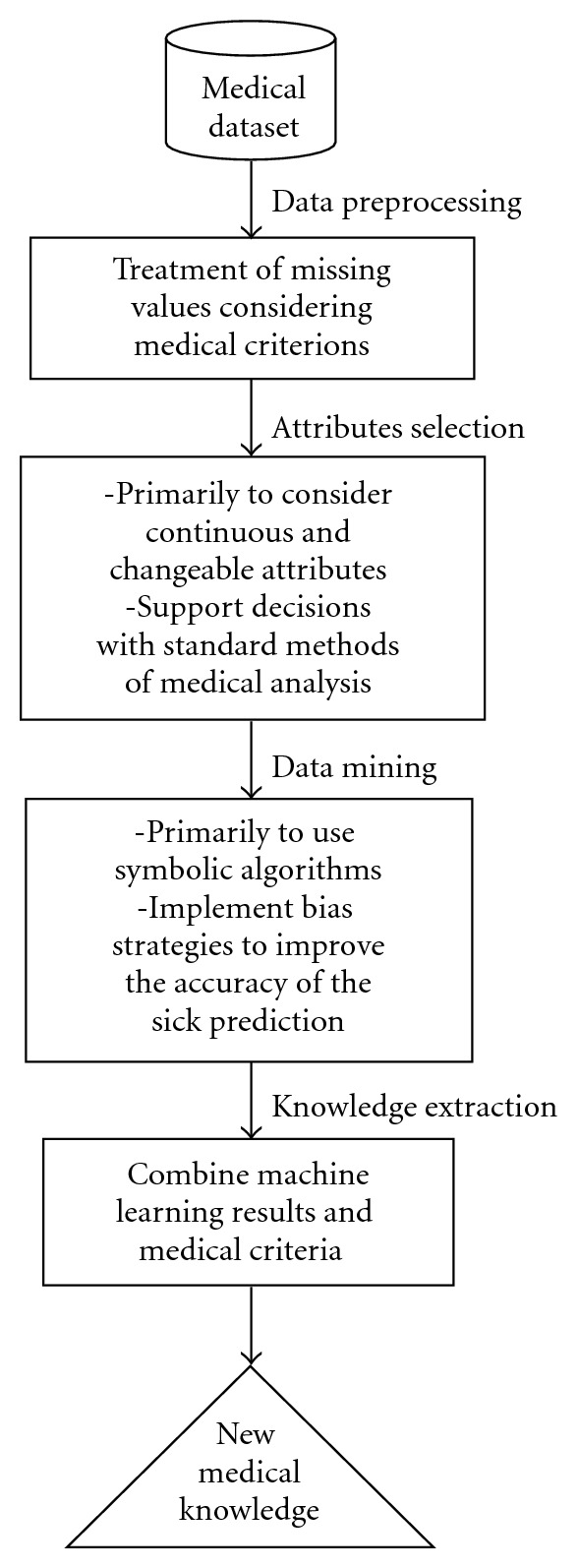
Machine learning method proposed.

We implemented an interdisciplinary ML method that encompassed all stages of knowledge extraction from databases (data preprocessing, attribute selection, data mining, and knowledge extraction), to examine the application of ML to support clinical decisions (Figure 2).

To improve the accuracy of predictions for affected subjects (positive class), we used the Rule Extraction for MEdical Diagnosis (REMED) algorithm [9], a symbolic one-class classification approach that implements internal bias strategies during the learning process [21]. REMED employs three main procedures in the knowledge extraction process: (1) selection of attributes, (2) selection of initial partitions, and (3) construction of classification rules.

First, REMED attempts to select the best combination of relevant attributes, using a simple logistic regression model. This is a standard method of analysis in medical research that uses the odds ratio metric [22] to determine if there is a significant association (*P* < 0.01) between a considered attribute and the positive class. REMED then begins to build initial partitions (exclusionary and exhaustive conditions) to maximize sensitivity and maintain acceptable accuracy without significantly decreasing specificity. Finally, REMED uses the respective partitions for each selected attribute to construct a system of rules that includes *m* conditions (one for each selected attribute) in the following way:

 
**If** Condition 1 <relation> *p*_1_
 
**and** Condition 2 <relation> *p*_2_
 
**and** Condition *j* <relation> *p*_*j*_ **and** *⋯⋯⋯*
 
**and** Condition *m* <relation> *p*_*m*_
 
**then** class = 1
 
**Else** class = 0,

where <relation> is either ≥ or ≤ depending on whether *j* is positively or negatively associated with the positive class through *p*_*j*_ (partition for attribute *j*).

To avoid overfitting during the training and testing phase, REMED implements the k-fold cross validation technique, which is based on randomly shuffling sample vectors among training and testing spaces [23]. REMED also maintains the approximate imbalance of the original dataset through the *k* iterations.

### 2.5. Data Preprocessing and Attributes Selection

Based on current medical guidelines [24], we only included participants that had ABP recordings of good technical quality. Therefore, subjects with <40 BP readings during the 24-hour ABP period were excluded. Systolic BP readings values >260 mmHg or <70 mmHg, and diastolic BP readings >150 mmHg or <40 mmHg were considered outliers or erroneous values and discarded. The treatment of missing values was addressed with predictive techniques, specifically multiple linear regression analysis considering medical criterions. 

Only continuous and changeable attributes were considered in the knowledge extraction process. Continuous attributes have a higher degree of uncertainty than discrete attributes, because discrete attributes are usually binary in the clinical environment (e.g., smoker versus nonsmoker), and their associations with specific diseases are almost always well known. We also excluded age, which is a nonchangeable attribute. The attributes considered in the initial ML analysis were body mass index (BMI), serum cholesterol level, 24-hour heart rate, and systolic and diastolic 24-hour WBP and ARV.

## 3. Results

### 3.1. Dataset

The minable dataset was composed of 551 observations with 7 attributes, with only 43 missing values (1.1%) in the serum cholesterol attribute. The missing data were estimated from the regression slope on sex and age, according to the criteria of physician specialists. The sample included 374 women (67.8%) and 170 patients (30.9%) undergoing treatment with antihypertensive drugs (Table 1). The average number of BP readings was 65.1 (5th to 95th percentile = 51.5−77.5), indicating good quality ABP recordings. Mean age was 67.1 ± 8 years. At enrolment, 61 participants (11.1%) had a history of cardiovascular disease; 100 (18.1%) had a history of diabetes mellitus, of whom 59 (59%) were undergoing diabetes treatment; 86 (15.6%) were current smokers; 174 (31.6%) reported intake of alcohol. The average total cholesterol level was 5.5 ± 1.3 mmol L^−1^, and BMI averaged 27.1 ± 5.6 kg m^−2^. Mean 24-hour systolic WBP was 133.8 ± 16.6 mmHg, and diastolic WBP was 76.1 ± 10 mmHg. Average heart rate was 73.7 ± 9.8 bpm.

The median follow-up period was 7.1 ± 3.7 years (5th to 95th percentile = 1.7−12.3 years). Only the participants that died from cardiovascular diseases (*n* = 61) were classified as positive examples. Cardiovascular mortality included 10 strokes and 51 cardiac deaths for a high event rate of 15.5 per 1000 person-years. The imbalance ratio between the positive (affected) and negative (unaffected) class was approximately of 1 : 9.

### 3.2. Machine Learning Process

#### 3.2.1. Selection of Attributes

Using the simple logistic regression model, REMED found only two attributes significantly associated with the positive class: systolic WBP (*P* = 0.008) and ARV (*P* = 0.0001). However, other well-known cardiovascular risk factors, such as serum cholesterol level, BMI, and diastolic WBP [16, 25], were considered in further analyses.

#### 3.2.2. Rule System

To provide medical staff with more information and comprehensible models, we used REMED to build several simple rule systems, which included individual and combined predictions of the more significant attributes (systolic WBP and ARV), as well as the combined predictions with the additional risk factors.

### 3.3. Performance

The confusion matrix from the predictions of the system rule, combining only high systolic ARV and WBP and using 10-fold cross-validation, indicated that REMED performed at 0.36 sensitivity, correctly diagnosing more than 35% of the cardiovascular deaths (Table 2). REMED focuses on improving sensitivity over specificity, because in the case of medical diagnosis/prognosis, the cost of misclassification of false negatives (FN, i.e., sick subjects diagnosed incorrectly) is higher than that of false positives (FP, healthy subjects diagnosed incorrectly), because more specific medical tests could discover the FP error, but an FN could cause a life-threatening condition and possibly lead to death [26]. Additionally, to compare the performance of our approach in terms of reliable prediction, we selected from the WEKA framework [2] the ML approach that better performed with our dataset: the Naïve Bayes classifier, which is one of the most effective and efficient classification algorithms and has been successfully applied to many medical problems [27, 28]. The performance of all classifiers is showed in Table 3.

## 4. Discussion

Use of the REMED algorithm selecting only the more significant attributes provided some of the desired features for solving medical diagnosis/prognosis problems: (1) good overall performance for imbalanced datasets, with 36.1% of sensitivity, 80% specificity, and 75.1% general accuracy; (2) comprehensible prognostic information, based on a rule system with a high degree of abstraction (only one rule to predict positive class examples, independent of the number of instances and initial attributes); (3) the ability to provide the medical staff with sufficient confidence to use the rule system in practice, because it was based on attributes with high confidence levels (>99%), estimated with a standard method of medical analysis; (4) the ability to reduce the number of medical tests necessary to obtain a reliable diagnosis/prognosis, because a simple logistic regression model was used to select attributes strongly associated with the specific disease.

The ML approach generated a new prognostic threshold for cardiovascular mortality: systolic WBP ≥ 137 mmHg, which is lower than the currently proposed by hypertension guidelines (≥140 mmHg) and in agreement with recent ABP studies [29, 30], but with the advantage that our analysis was fully automated and had a smaller sample. Moreover, our ML approach generated a new prognostic threshold for abnormal systolic ARV (≥9.6 mmHg). Together, these new thresholds could provide improved predictions of cardiovascular mortality.

Both systolic WBP and ARV were independent predictors of cardiovascular mortality, performed >50% of sensitivity, but sacrificed significantly in specificity and general accuracy (≤60%). The addition of other well-known cardiovascular risk factors decreased considerably the accuracy in the prediction of affected subjects (<23%). Therefore, the use of logistic regression for the selection of significant attributes (>99%) could be an effective strategy in this stage of ML analysis in medical datasets.

Undoubtedly, one of the most important goals of the application of ML in the medical field is to generate new knowledge, providing the medical community with tools to develop novel points of view about any given problem. In our case, for example, although previous medical studies determined possible ranges of a low and high BPV measured whit ARV through statistical methods (median and quartiles analysis) [17, 18], our work is pioneer proposing a prognostic threshold for abnormal systolic ARV (≥9.6 mmHg). This threshold has a good performance as an independent a composed predictor of fatal cardiovascular events. The use of this threshold should facilitate new fields of investigation regarding BPV and its prognostic relevance.

We do not claim that our ML analysis using REMED is the ultimate solution for medical diagnosis/prognosis problems from unbalanced datasets, because it is necessary to implement modifications that improve REMED's predictive capacity in terms of sensitivity (≥50%) without significantly deteriorating its specificity. However, we obtained better results than the Naïve Bayes classifier (11.48%), which is considered as a benchmark algorithm that in any medical domain has to be tried before any other advanced method [27]. Therefore, we believe that our approach could improve performance in these medical tasks, and increase the confidence of the medical community in the use of ML approaches to support clinical decisions.

## Figures and Tables

**Table 1:** Baseline characteristics.

	Frequency in percent or median
Demographic variables	
Men, % (*n*)	32.1 (177)
Age, years	67.1 ± 8
Race, % (*n*)	
Mixed	73.1 (404)
Caucasian	22.2 (122)
African-Venezuelan	4 (22)
Natives	0.5 (3)
Use of antihypertensive drugs, % (*n*)	30.9 (170)
Use of anti-diabetic drugs, % (*n*)	11.1 (61)
History of cardiovascular disease, % (*n*)	11.5 (63)
Diagnosis of diabetes mellitus, % (*n*)	18.1 (100)
Lifestyle, physical and lipid factors	
Smoking current status, % (*n*)	15.6 (86)
Drinking current status, % (*n*)	31.6 (174)
Body max index, kg/m^2^	27.1 ± 5.6
Total serum cholesterol, mmol/L	5.5 ± 1.3
24-hour ambulatory measurements	
Systolic blood pressure, mm Hg	133.8 ± 16.6
Diastolic blood pressure, mm Hg	76.1 ± 10
Heart rate, bpm	73.7 ± 9.8

**Table 2:** Confusion matrix of REMED predictions.

		Predictive class	
		Positive	Negative
Actual class	Positive	22	39
	Negative	98	392

**Table 3:** Performance of classifiers.

Classifiers	Sensitivity	Specificity	Accuracy
**I** **f** systolic ARV ≥ 9.6 **then** 1 **Else** 0	55.7%	60.4%	59.9%
**If** systolic WBP ≥ 134.6 **then** 1 **Else** 0	52.5%	58.8%	58.08%
**If** systolic ARV ≥ 9.6 **and** systolic WBP ≥ 137 **then** 1 **Else** 0	36.1%	80.0%	75.1%
**If** systolic ARV ≥ 9.6 **and **systolic WBP ≥ 138.6 **and** cholesterol ≥ 5.5 **then** 1 **Else** 0	8.2%	93.3%	83.8%
**If** systolic ARV ≥ 10.4 **and **systolic WBP ≥ 139.8 **and** BMI ≥ 27.3 **then** 1 **Else** 0	9.8%	93.3%	84.0%
**If** systolic ARV ≥ 9.6 **and **systolic WBP ≥ 137 **and** diastolic WBP ≥ 78.4 **then** 1 **Else** 0	22.9%	87.5%	80.4%
**Naïve Bayes**	11.48%	95.92%	86.57%
